# *Bifidobacterium breve* synergizes with *Akkermansia muciniphila* and *Bacteroides ovatus* to antagonize *Clostridioides difficile*

**DOI:** 10.1093/ismejo/wraf086

**Published:** 2025-04-30

**Authors:** Yanan Li, Wen Rui, Xiaoya Sheng, Xilong Deng, Xiaoqian Li, Lingtong Meng, He Huang, Jingpeng Yang

**Affiliations:** State Key Laboratory of Microbial Technology, Nanjing Normal University, 2 Xuelin Road, Qixia District, Nanjing, Jiangsu 210033, China; School of Food Science and Pharmaceutical Engineering, Nanjing Normal University, 2 Xuelin Road, Qixia District, Nanjing, Jiangsu 210033, China; State Key Laboratory of Microbial Technology, Nanjing Normal University, 2 Xuelin Road, Qixia District, Nanjing, Jiangsu 210033, China; School of Food Science and Pharmaceutical Engineering, Nanjing Normal University, 2 Xuelin Road, Qixia District, Nanjing, Jiangsu 210033, China; State Key Laboratory of Microbial Technology, Nanjing Normal University, 2 Xuelin Road, Qixia District, Nanjing, Jiangsu 210033, China; School of Food Science and Pharmaceutical Engineering, Nanjing Normal University, 2 Xuelin Road, Qixia District, Nanjing, Jiangsu 210033, China; State Key Laboratory of Microbial Technology, Nanjing Normal University, 2 Xuelin Road, Qixia District, Nanjing, Jiangsu 210033, China; School of Food Science and Pharmaceutical Engineering, Nanjing Normal University, 2 Xuelin Road, Qixia District, Nanjing, Jiangsu 210033, China; State Key Laboratory of Microbial Technology, Nanjing Normal University, 2 Xuelin Road, Qixia District, Nanjing, Jiangsu 210033, China; School of Food Science and Pharmaceutical Engineering, Nanjing Normal University, 2 Xuelin Road, Qixia District, Nanjing, Jiangsu 210033, China; State Key Laboratory of Microbial Technology, Nanjing Normal University, 2 Xuelin Road, Qixia District, Nanjing, Jiangsu 210033, China; School of Food Science and Pharmaceutical Engineering, Nanjing Normal University, 2 Xuelin Road, Qixia District, Nanjing, Jiangsu 210033, China; State Key Laboratory of Microbial Technology, Nanjing Normal University, 2 Xuelin Road, Qixia District, Nanjing, Jiangsu 210033, China; School of Food Science and Pharmaceutical Engineering, Nanjing Normal University, 2 Xuelin Road, Qixia District, Nanjing, Jiangsu 210033, China; State Key Laboratory of Microbial Technology, Nanjing Normal University, 2 Xuelin Road, Qixia District, Nanjing, Jiangsu 210033, China; School of Food Science and Pharmaceutical Engineering, Nanjing Normal University, 2 Xuelin Road, Qixia District, Nanjing, Jiangsu 210033, China

**Keywords:** Clostridioides difficile, *Akkermansia muciniphila*, *Bacteroides ovatus*, *Bifidobacterium breve*commensalism, NF-κB signaling pathway

## Abstract

The development of ecologically based *in vivo* microecological formulations for treating Clostridioides difficile infection (CDI) is a current research focus. Here, we selected three microorganisms—*Akkermansia muciniphila* (AM), *Bacteroides ovatus* (BO), and *Bifidobacterium breve* (BB)—to formulate a mixed bacterial formulation (ABB). Subsequently, we evaluated the ecological interactions among these three microorganisms and investigated their therapeutic efficacy in a CDI murine model. Our investigation revealed the presence of a commensalism relationship among AM, BO, and BB. These microorganisms collectively formed a robust and densely packed symbiotic biofilm, with BB being the predominant member in terms of numerical abundance. This phenomenon was concomitant with a marked elevation in the levels of AI-2 and c-di-GMP. ABB exhibits the capability to inhibit crucial biological indicators of *C. difficile* (CD), such as toxin production, through the secretion of substantial quantities of lactic acid. Additionally, ABB indirectly suppresses CD by activating the NF-κB signaling pathway in Raw 264.7 cells, which stimulates the secretion of significant quantities of IL-6, IL-8, TNF-α, and IL-1β. ABB demonstrated exceptional efficacy in a CDI murine model, as evidenced by a substantial enhancement in survival rates and intestinal short-chain fatty acid level, the down-regulation of inflammation-associated cytokine secretion, a notable reduction in fecal CD toxin levels, and CD viable bacterial counts. Concurrently, there was an augmentation in the level of gut microbial diversity, accompanied by a rapid reduction in Enterococcus abundance. This ABB formulation holds promise for further development into a novel microecological formulation for the treatment of CDI.

## Introduction

*Clostridioides difficile* (CD), a resident member of the human gut microbiota, can cause intestinal infections under specific conditions, such as excessive antibiotic use, a condition known as *C. difficile* infection (CDI) [1]. Primary *C. difficile* infection (pCDI) is typically managed with vancomycin (VAN) and fidaxomicin, although these treatments are associated with a certain recurrence rate (20%–25%) [2]. Recurrent *C. difficile* infection (rCDI) is frequently treated using fecal microbiota transplantation (FMT) [3]. Based on clinical outcomes, a small percentage of patients exhibit non-response to treatment with either pCDI or rCDI. This phenomenon can be attributed to several factors, including the presence of antibiotic-resistance genes in CD, the persistence of recalcitrant endospores, and the presence of harmful companion members in the gut, such as Enterococcaceae and Enterobacteriaceae [2, 4]. Indeed, the primary issue with CDI centers on alterations in the host's gut ecosystem, encompassing shifts in the composition and concentration of gut nutrients and microbiota [5]. The ecological interactions among gut microbiota members significantly impact the severity of infections or the effectiveness of treatments [6]. Specifically, *Enterococcus faecalis*, *Fusobacterium nucleatum*, and *Candida albicans* can form symbiotic biofilms with the CD or supply it with fermentable amino acids, thereby enhancing CD toxin production and exacerbating co-infections and inflammatory responses [6–8]. Conversely, intestinal Bifidobacterium, Bacteroides, Lactobacillus, and Akkermansia have been shown to compete with CD for ecological niches and secrete antimicrobial substances, which inhibit CD colonization, expansion, and toxin production [9]. Consequently, developing strategies to prevent, mitigate, or treat CDI through gut ecological regulation may prove more effective. This perspective has gained considerable recognition within the scientific community, particularly among clinical microbiologists and infectious disease specialists. Orally administered microbial consortia specifically developed for rCDI management, including SER-109 and VE303 formulations, have demonstrated promising clinical efficacy in randomized controlled trials [10, 11]. These microbiota-based therapeutics are currently emerging as frontrunners in the field of microbiome restoration therapy.

Based on multiple published clinical studies, we observed a significant reduction in the abundance of the genus-level bacteria Akkermansia, Bifidobacterium, and Bacteroides during CDI, which exhibited a strong negative correlation with intestinal inflammation [5, 12–14]. Our prior research indicated that microbial community structure was markedly disrupted during infection, primarily characterized by a substantial decline in the abundance of specific members such as *A. muciniphila* (AM), *B. breve* (BB), and *B. ovatus* (BO) [15]. Consequently, in this study, these three microorganisms—AM, BB, and BO—were selected as target strains to explore their ecological interactions in vitro and the antagonistic effects of the combined bacterial formulation (ABB) on CD. Using a CDI murine model, we assessed the therapeutic efficacy of ABB and analyzed the changes in the gut microbiota of mice under various treatments, providing a foundation for the further development of this mixed-bacterial formulation.

## Materials and methods

### Bacterial strains and cell lines

Clostridioides difficile ATCC 43255 (CD) and *Vibrio campbellii* ATCC BAA-1117 (VC) were obtained from the American Type Culture Collection (ATCC; Manassas, VA, USA). AM JP101, BO JP106, and BB ZZ409 (CCTCC M 20241663, BB) are maintained and provided by our laboratory. AM, BO, and BB collectively form a three-bacterial microecological preparation named ABB. For cultivation, CD and AM were grown in Brain Heart Infusion (BHI) broth, BO in Tryptic Soy Broth (TSB) broth, and BB in de Man Rogosa Sharpe (MRS) broth. All strains were activated and incubated in an anaerobic workstation (85% nitrogen, 10% hydrogen, 5% carbon dioxide, Electrotek Anaerobic Workstation, AW 400TG Two Gas Version, United Kingdom) at 37°C except VC. VC cultured in Autoinducer Bioassay (AB) broth at 30°C. The mouse monocyte–macrophage Raw 264.7 cell line, maintained and provided by our laboratory, was cultured in a complete Dulbecco's Modified Eagle's Medium (DMEM). This complete medium comprised DMEM high-glucose medium (supplemented with 100 IU/ml penicillin and 100 μg/ml streptomycin, without dual antibiotics; KGI Bio, Jiangsu, China) and 10% (v/v) fetal bovine serum (Gibco, Jiangsu, China).

### Ecological interactions among two of the three microorganisms

AM, BO, and BB were sequentially passaged and gradually adapted to BHI culture. Formal experiments commenced once all strains exhibited robust growth in BHI broth. Bacterial suspensions of AM, BO, and BB, with nearly identical CFU values (10^9^ CFU/ml) during the logarithmic phase, were prepared individually. Following centrifugation and filtered through a sterile filter (0.22 μm), cell-free culture supernatants (CFCS) and bacterial cell pellets (cells) were collected. The bacterial cells from each strain were resuspended to achieve 10^9^ CFU/ml. The bacterial suspensions were then mixed with CFCS at a volume ratio of 1: 2. The growth, biofilm formation, and motility of both individual and co-cultured bacteria were evaluated [16]. For the growth determination, the different combinations were placed in 96-well plates, and after anaerobic incubation at 37°C for 48 h, OD_630nm_ was determined by a microplate reader (H1M, BioTek, USA). The control group was replaced with an equal volume of fresh BHI broth. For the biofilm determination, the different combinations were placed in 96-well plates, and after anaerobic incubation at 37°C for 72 h, the bacterial cells were removed, and then crystal violet staining was used to evaluate the biofilm (OD_570nm_). For the bacterial motility determination, the different combinations were anaerobic and incubated at 37°C for 12 h. After that, the bacterial solution was punctured and inoculated on 1/2BHI semi-solid plates (0.5% agar), which were anaerobically incubated at 37°C for 4 d. Colony diameter was measured. The control group was replaced with an equal volume of fresh BHI broth.

### Fluorescence in situ hybridization coupled with scanning electron microscopy was utilized to evaluate the ecological distribution of three bacterial members following co-culture

AM, BO, and BB were cultured to a stable phase, and their cell concentrations were adjusted to a similar level (10^9^ CFU/ml). They were then combined in various ratios. The monoculture systems consisted of 2 ml of AM, BO, or BB, each mixed with 4 ml of fresh BHI broth. The dual bacterial systems included 2 ml of AM +2 ml of BO +2 ml of fresh BHI broth, 2 ml of AM +2 ml of BB + 2 ml of fresh BHI broth, and 2 ml of BO +2 ml of BB + 2 ml of fresh BHI broth. The triple bacterial system comprised 2 ml of AM +2 ml of BO +2 ml of BB. Each group was subsequently supplemented with 8 ml of fresh BHI broth and incubated in an anaerobic workstation at 37°C for 24 h. The bacterial cells from each group were harvested and washed with PBS, followed by fixation with 4% paraformaldehyde solution at 4°C for 12 h. After washing with PBS, the samples were transferred to a 50% ethanol solution in PBS and fixed at 4°C for an additional 12 h.

100 ng of each of the three specific probes was combined with 20 μl of hybridization solution (6 × SSC, 5 × Denhardt's solution, 0.5% SDS, 100 μg/ml salmon sperm DNA, 50% formamide). The resulting mixture was added to the bacterial cells and incubated at 46°C for 2 h. Subsequently, the unbound probes were removed by repeated washing with a wash buffer (20 mM Tris/HCl, 5 mM EDTA, 159 mM NaCl, 0.01% (w/v) SDS, pH 7.5) at 48°C for 15 min. The distribution of bacteria in the different groups was visualized using a laser confocal scanning microscope (AX Confocal Microscope System, Nikon, Japan). The fluorescence in situ hybridization (FISH) probes (Sangon Biotech, Shanghai, China) used in this study were as follows: AM-Specific probes, 5′- CCT TGC GGT TGG CTT CAG AT −3′, 5′ modified with Cy3, Ex/Em = 550/570 nm [17–19]. BO-Specific probes, 5′- CCT TCA CAA CAG CCT TAC G − 3′, 5′ modified with Cy5, Ex/Em = 640/670 nm [20]. BB-Specific probes, 5′- CCA TGC GGT GTG ATG GAG C -3′, 5′ modified with 6-FAM, Ex/Em = 494/518 nm [21]. BB-Assisted probes, PBR2-1^st^-Upper helper, 5′- ATC CGG CAT TAC CAC CCG T -3′; PBR2-1^st^-Lower helper, 5′- CAA AGG CTT TCC CAA CAC A -3′; PBR2-2^nd^-Upper helper, 5′- TTC CAG GAG CTA TTC CGG T -3′; PBR2-2^nd^-Lower helper, 5′- GCG ACC CCA TCC CAT GCC G -3′ [21].

The co-culture of the three bacteria was conducted using an identical methodology. Following an anaerobic incubation at 37°C for 24 h, the single and mixed bacterial cells were washed once with PBS. Subsequently, a 2.5% glutaraldehyde solution was added to fix the cellular morphology of the bacterial cells, which was maintained overnight at 4°C. Gradient dehydration was performed using 25%, 50%, 75%, 95%, and 100% ethanol, with each step lasting 15 min and repeated twice. The samples were then freeze-dried at −60°C and 0 Pa in a vacuum freeze-dryer (Sihuan Qihang Technology, Beijing, China). After gold coating, the samples were examined under a scanning electron microscope (SU8010, HITACHI, Japan), and images were captured.

### SEM in conjunction with confocal laser scanning microscopy was utilized to evaluate the ecological distribution of microorganisms within the symbiotic biofilms

Sterile circular coverslips were placed at the bottom of 12-well plates. AM, BO, and BB were cultured until the late logarithmic phase, and the bacterial cell concentrations were adjusted to be similar (10^9^ CFU/ml). For the three-bacteria system, 100 μl each of AM, BO, and BB were added to the 12-well plates, and fresh BHI was added to achieve a total culture volume of 3 ml, which was then incubated for 48 h. After incubation, the biofilm was washed once with PBS, and the bacterial cells were transferred to 24-well plates containing 2 ml of 2.5% glutaraldehyde solution to fix their morphology overnight at 4°C. Subsequently, the samples underwent gradient dehydration using 25%, 50%, 75%, 95%, and 100% ethanol, with each step lasting 15 min and repeated twice. Subsequently, the samples were freeze-dried at −60°C and 0 Pa using a vacuum freeze-dryer (Sihuan Qihang Technology, Beijing, China). Following gold sputtering, the samples were imaged using a scanning electron microscope (Apreo 2S, Thermo Scientific, USA), and the images were captured with Inletex Easy Meeting Classic Pro software. The same procedure was used to cultivate dual and triple bacterial biofilms. The formed biofilms were washed with PBS, and SYTO Green (KGI Bio, Nanjing, China) was diluted 2000-fold with fresh BHI and added to the samples. The samples were incubated in the dark for 1 h, washed again with PBS, and then covered with 20 mm × 20 mm coverslips with the biofilm side facing down. The samples were then examined under a confocal laser scanning microscope (AX Confocal Microscope System, Nikon, Japan) equipped with a × 60 oil-immersion lens, and the biofilm was scanned in the Z-axis direction (Ex/Em = 500/530 nm). For the hybridization experiments, the biofilms were washed with PBS, and AM, BO, and BB-specific probes were added. The distribution of the triple bacterial cells in the symbiotic biofilm was observed using a confocal laser scanning microscope (AX Confocal Microscope System, Nikon, Japan).

### Determination of three microorganisms in symbiotic biofilms and murine fecal samples by qPCR

Absolute quantification of each microbe in the symbiotic biofilm and the murine fecal samples was performed by qPCR. Murine fecal samples at T2 and T3 were collected separately, homogenized, and resuspended in sterile PBS. The specific steps were as follows: firstly, the DNA of AM, BO and BB were extracted as templates and sent to Sangon Biotech Co., Ltd (Shanghai, China); secondly, TA cloning and sequencing were carried out according to the specific primers, and the plasmids (1865 bp) containing the target genes (Table S1) were extracted as the standards for the preparation of the standard curves; and finally, the AM, BO and BB in the symbiotic biofilm and the murine fecal samples were quantified according to the Ct value and the copy number. Copies/μl = (DNA concentration (ng/μl) × 6.02 × 10^14^/ [660 × (plasmid length + target gene)]. AM-specific primers: AMF-CAGCACGTGAAGGTGGGGAC, AMR-CCTTGCGGTTGGCTTCAGAT [22]; BB-specific primers: BBF-CCGGATGCTCCATCACAC, BBR-ACAAAGTGCCTTGCTCCCT [23, 24]. BO-specific primers: BOF-CCGGATAGCATACGAAYAT, BOR-CACAACTGACTTAACAATCC [25].

### Determination of AI-2 and c-di-GMP levels in biofilm systems

The bacterial monophyletic biofilm and mixed symbiotic biofilm culture solutions were collected and centrifuged, and the supernatants were collected. The AI-2 contents in the mono or symbiotic biofilm systems were determined according to a previously published method with some modifications [26]. The VC bacteria solution cultured overnight was diluted 500 times with fresh AB broth. After that, the bacterial monophyletic biofilm and mixed symbiotic biofilm culture solutions CFCS was mixed with the diluted VC bacteria solution at the volume ratio of 1:10, respectively, and incubated at 30°C. The fluorescence intensity of each sample was measured by a microplate reader (H1M, BioTek, USA) with the lowest fluorescence intensity of the control group (without monophyletic biofilm and mixed symbiotic biofilm culture solutions CFCS), and the relative fluorescence intensity was calculated, representing the activity of the signal molecule AI-2. Fifty microliters of the CFCS corresponding to the bacterial monophyletic biofilm and mixed symbiotic biofilm culture solutions were taken, and the content of cyclic di-GMP (c-di-GMP) in the supernatant was determined by using a Bacterial c-di-GMP ELISA kit (LMAI Bio, Shanghai, China) [16].

### Direct killing effects of ABB on *C. difficile* growth, biofilm production, and toxin protein production were assessed based on the OD assay and enzyme-linked immunoassay

Bacterial solutions from the late logarithmic growth phases of AM, BO, and BB were harvested and centrifuged to isolate the bacterial cells (cells) and CFCSs. The isolated cells from the three strains were resuspended in a fresh BHI broth to create bacterial suspensions with comparable OD_600nm_ values (10^9^ CFU/ml). A fresh CD bacterial suspension (2.5 × 10^8^ CFU/ml) was also prepared. Subsequently, the cells and CFCS from the different microorganisms were co-cultured with CD cells using Transwell chambers (6.5 mm diameter inserts, 0.4 μm pore size, polycarbonate membrane, Corning, USA). After 24 to 48 h, the changes in CD growth and biofilm formation were measured by a microplate reader (H1M, BioTek, USA), and the levels of toxin proteins TcdA and TcdB were assessed using the CD TOXA/B II kit (Tech Lab, Kraft Drive, Blacksburg, VA, USA).

### Analysis of the main antibacterial substances of ABB CFCS

Microorganisms secrete a variety of metabolites, among which the main substances that can act as antibacterials are organic acids, hydrogen peroxide, bacteriocins, and antimicrobial peptides. Using the Oxford cup method, we separately analyzed the main antibacterial substances in ABB-CFCS [26]. For organic acid analysis, NaOH solution (1 mol/L) was used to adjust the pH of ABB-CFCS to 7, and then an antibacterial test was performed, in which ABB-CFCS without pH adjustment was used as a control. For hydrogen peroxide analysis, 10 mg/ml hydrogen peroxide solution was added to ABB-CFCS, and the antibacterial test was performed after a water bath at 35°C for 2 h, where ABB-CFCS without hydrogen peroxide was used as a control. For protein-based antimicrobial substance analysis, pepsin, trypsin, and proteinase K (Sangon Biotech, Shanghai, China) were added to ABB-CFCS, and the concentration was adjusted to 1 mg/ml. The enzymes were incubated in a water bath at 37°C for 2 h and inactivated at 80°C for 2 min, after which an inhibition test was performed, in which ABB-CFCS without protease treatment was used as the control. For thermal stability analysis, ABB-CFCS was subjected to 60, 80, and 100°C and a water bath for 5 min for an antibacterial test to evaluate the thermal stability of the inhibitory substances, in which untreated ABB-CFCS was used as a control.

### Types and content of organic acids in ABB-CFCS

To identify the main antibacterial components in the CFCS of both single and mixed bacterial formulations (ABB), which were found to be organic acids, liquid chromatography–tandem mass spectrometry (LC–MS/MS) was employed to determine the types and concentrations of organic acids in the fermentation supernatants of both individual (10^9^ CFU/ml) and ABB (10^9^ CFU/ml), with fresh BHI broth serving as the control. The chromatographic and mass spectrometric conditions were based on a previously published method [26].

### Indirect killing effect of cytokines secreted by ABB co-culture with raw 264.7 on CD was evaluated by enzyme-linked immunoassay

ABB was conducted following the methods of previous studies, incubated at 37°C in an anaerobic workstation for 24 hours, centrifuged, and the supernatants discarded [27]. The mixed bacterial cell pellets were washed three times with PBS and resuspended in an antibiotic-free DMEM medium. Raw 264.7 cells were grown to 70% in triplicate at 37°C (5% CO_2_) and resuspended in a cell culture medium, with the number of cells per replicate adjusted to 5 × 10^5^ cells per ml [28]. Afterward, one copy was used as a negative control (without the addition of lipopolysaccharide, LPS), one copy was added with LPS (100 ng/ml, sigma LPS L2880, originating from *E. coli* 055: B5) as a positive control, and one copy was added with LPS and ABB bacterial preparation. All treatments were placed at 37°C (5% CO_2_) for 24 h of incubation. After centrifugation, the supernatants of all treatments were filtered through a 0.22 μm filter membrane, and the levels of IL-1β, TNF-α, IL-6, and IL-8 in these supernatants were quantified using corresponding cytokine ELISA kits (Enzyme-Linked Bio, Shanghai, China). Additionally, using the broth double dilution method, the supernatant was serially diluted with fresh BHI broth in a 96-well plate, and 100 μl of CD bacterial suspension (5 × 10^7^ CFU/ml) was added to each well. Absorbance was measured using a microplate reader (H1M, BioTek, USA) after incubation in an anaerobic workstation at 37°C for 48 h.

#### Western blot

Further verification confirmed that the ABB formulation exerts an indirect bactericidal effect by activating the TLR4/NF-κB pathway in Raw 264.7 cells. Raw 264.7 cells were cultured in DMEM complete medium until reaching approximately 70% confluence. The medium was then discarded, and the cells were washed three times with PBS. Subsequently, the cells were co-cultured with mono- (AM, BO, BB) or triple-bacterial co-cultures (ABB) (previously resuspended in antibiotic-free DMEM medium). After 24 h, the cells were washed with pre-cooled PBS, scraped off using a spatula, and resuspended in pre-cooled PBS. The collected Raw 264.7 cells were lysed with Western and IP cell lysis buffer (KGI Bio, Jiangsu, China) to extract proteins. Protein concentrations were determined using a BCA protein assay kit (KGI Bio, Jiangsu, China). The proteins were separated on an 8% SDS-polyacrylamide gel (SDS-PAGE Gel Configuration Kit, KGI Bio, Jiangsu, China) and transferred to a PVDF membrane, which was blocked with 5% skimmed milk for 1 h. The membrane was incubated overnight at 4°C with primary antibodies against NF-κB p65 (A19653, ABclonal, USA, 1:1000), IκBα (AF5002, Affinity Biosciences, China, 1:1000), and TLR4 (AF7017, Affinity Biosciences, China, 1:1000). Following this, the membrane was incubated with anti-rabbit IgG-HRP secondary antibody (7074P2, CST, USA, 1:3000) for 2 h before developing. β-Actin antibody (20536–1-AP, Proteintech, China, 1:3000) served as the internal reference protein. The Raw 264.7 cells without LPS stimulation were included as the negative control.

#### CDI mouse model

Fifty-five 6- to 8-week-old male C57BL/6 mice were purchased from Shanghai SLAC Laboratory Animal, Co., Ltd. (Shanghai, China) and reared in the Animal Laboratory Center of Nanjing Normal University. All these mice acclimatized for one week before the commencement of the experiment. The CDI mouse model was established following a previously published method [29]. Mixed antibiotic treatment (0.14 mg/ml gentamicin, 1.6 mg/ml kanamycin, 0.86 mg/ml metronidazole, 0.168 mg/ml colistin, and 0.18 mg/ml VAN; Macklin, Shanghai, China) was used for 5 d, after which each mouse was gavaged with CD bacterial suspension (10^8^ CFU/100 μl/each) [30]. There were 6 treatment groups, which were started at the first sign of infection (loose stools, sudden weight loss). Among them, the NC group (n = 5) grew normally without any treatment throughout the whole process; the CD group (n = 10) received only PBS gavage (150 μl/each/times) after infection; the AM group (n = 10) was gavaged with AM (10^9^ CFU/150 μl/each/times) after infection, the BB group (n = 10) was gavaged with BB (10^9^ CFU/150 μl/each/times) after infection, and the BO group (n = 10) was gavaged with BO (10^9^ CFU/150 μl/each/times) after infection; ABB group (n = 10), AM, BO, and BB (10^9^ CFU/150 μl/each/times, 50 μl of each of the three bacterial solutions) were gavaged after infection. Mice were monitored for survival and body weight throughout the experiment and were euthanized if they approached death or experienced a body weight loss exceeding 20% from baseline. The bacterial solutions (AM, BO, BB, and ABB) were administered every 24 h until the mice returned to a state comparable to pre-infection levels (including body weight, and absence of diarrhea symptoms), at which point administration ceased. Subsequently, all mice underwent euthanasia after 7 d of normal feeding and observation. Upon apparent death during the experiment, cecum-colonic tissues were immediately collected, and the cecum-colonic length was measured and preserved in a 10% formalin solution. Additionally, mouse fecal samples were collected at three designated time points and stored in sterile tubes at −80°C. Fecal samples were collected at the following time points: before infection (Time 1, T1), at the onset of drug administration (Time 2, T2), and after drug administration (Time 3, T3). Following the final sampling point (T3), after an additional week of observation, the surviving mice were euthanized, and their cecum tissues were collected for hematoxylin and eosin (H&E) staining and immunohistochemistry (IHC). This animal experiment was approved by the Animal Ethical and Welfare Committee of Nanjing Normal University under the ethical number IACUC-20230253.

### H&E staining and cytokine measurements

The cecum tissues from mice in each group were processed for paraffin embedding and sectioning using a pathology microtome (Leica RM2016, Leica, Munich, Germany). Subsequently, the sections were stained with hematoxylin (Servicebio, Wuhan, China) for 5 min, followed by dehydration in 95% ethanol for 1 min. The sections were then stained with eosin (Servicebio, Wuhan, China) for 15 s, dehydrated, and mounted. Finally, the sections were examined and scanned using a microscope (Nikon E100, Tokyo, Japan) equipped with a digital camera (Nikon DS-U3, Tokyo, Japan). Colon tissues (20 mg) of mice at T3 were homogenized in RIPA buffer (containing protease inhibitors) (Santa Cruz Biotechnology, Santa Cruz, CA, USA), and then the supernatant was collected by centrifugation (12 000 × g for 10 min at 4°C) and the levels of IL-1β, TNF-α, IL-6, and IL-8 in these supernatants were quantified using corresponding cytokine ELISA kits (Enzyme-Linked Bio, Shanghai, China).

### Immunohistochemistry

The cecal tissues from each group of mice were processed for paraffin embedding and sectioning using a Leica RM2016 microtome (Leica, Munich, Germany). Subsequently, the sections were incubated in a 3% hydrogen peroxide solution (Anjie Gaoke, Shandong, China) for 25 min at room temperature in the dark to inhibit endogenous peroxidase activity. A 3% BSA solution (Servicebio, Wuhan, China) was applied to the histochemical circles to evenly cover the tissues and incubated for 30 min at room temperature for blocking. After gently removing the blocking solution, the sections were incubated with primary antibodies: Muc2 (Q02817, Servicebio, China), ZO-1 (Q07157, Servicebio, China), Occludin (Q16625, Servicebio, China), NF-κB p65 (A19653, ABclonal, USA), IκBα (AF5002, Affinity Biosciences, China), and TLR4 (AF7017, Affinity Biosciences, China) overnight at 4°C in a humidified chamber. The next day, the sections were incubated with an anti-rabbit IgG-HRP secondary antibody (Q9JMG7, Servicebio, China) for 50 minutes at room temperature. Freshly prepared DAB chromogen solution (Servicebio, Wuhan, China) was added, and the color development was monitored under a microscope until a brownish-yellow color appeared, after which the reaction was stopped by washing the sections with tap water. The nuclei were counterstained with hematoxylin (Servicebio, Wuhan, China) for 3 min. The sections were then dehydrated, cleared, and mounted. Finally, the slides were examined and scanned using a Nikon E100 microscope (Tokyo, Japan) equipped with a Nikon DS-U3 digital camera (Tokyo, Japan). ImageJ (version Fiji) software was used to convert the coloring depth and distribution area of target proteins into average optical density values to determine the expression of target proteins. The integrated option density (IOD) and area of each image were measured, and then the average optical density (AOD) was calculated to reflect the concentration of the target protein per unit area and AOD=IOD/Area. AOD of three random regions in each group was selected. The AOD of the areas was statistically analyzed using GraphPad Prism 10.

### Determination of CD numbers and toxin levels in mouse fecal based on CD moxalactam norfloxacin agar medium and enzyme immunoassay, respectively

Mouse fecal at T2 and T3 were collected separately, homogenized, and resuspended in sterile PBS in duplicate. One of the samples was gradient diluted, coated on CD moxalactam norfloxacin agar (Oxoid, Basingstoke, Hants, UK), and incubated at 37°C in an anaerobic workstation for 5 d for counting. The other sample was tested for toxin protein level through a CD TOXA/B II TM kit (Tech Lab, Kraft Drive Blacksburg, VA, USA).

### Abundance of AM, BO, and BB in mouse fecal was evaluated using quantitative PCR

Fecal samples from mice, collected from the preceding experimental step, were subjected to analysis. Drawing upon the established method for the absolute quantification of bacteria within symbiotic biofilms, quantitative PCR (qPCR) was employed to ascertain the copy numbers of AM, BO, and BB present in the mouse feces.

### Mouse fecal microbiome

Fecal total genomic DNA was extracted using an OMEGA Soil DNA kit (D5625–01, Omega Bio-Tek, Norcross, GA, USA) and stored at −20°C for further analysis. DNA was quantified using a NanoDrop ND-1000 spectrophotometer (Thermo Fisher Scientific, Waltham, MA, USA), and the quality of DNA extraction was checked by 1.2% agarose gel electrophoresis. PCR amplification was performed using Pfu high-fidelity DNA polymerase from All-Style Gold, and the number of amplification cycles was strictly controlled to keep the number of cycles as low as possible and to ensure that the amplification conditions of the same batch of samples were consistent. Negative controls are also set up to detect microbial contamination of the environment and reagents. Any sample population with bands amplified by the negative control may not be used in subsequent experiments. The PCR system was as follows: 5 μl of buffer (5×), 0.25 μl of Fast pfu DNA Polymerase (5 U/μl), dNTPs (2 μl, 2.5 mM), 1 μl each of pre and post-primers (10 μM), 1 μl of DNA template, 14.75 μl of ddH2O. Reaction conditions were 98°C for 5 min, followed by 25 cycles (including 30 s at 98°C, annealing at 53°C for 30 s, extension at 72°C for 45 s, and final extension at 72°C for 5 min). The reaction conditions were 5 min at 98°C, followed by 25 cycles (including 30 s at 98°C, annealing at 53°C for 30 s, extension at 72°C for 45 s, and final extension at 72°C for 5 min). PCR amplification products were purified with Vazyme VAHTSTM DNA Clean Beads (Vazyme, Nanjing, China) and quantified with Quant-iT PicoGreen dsDNA Assay Kit (Invitrogen, Carlsbad, CA, USA). Double-end sequencing was performed using the MiSeq System (Illumina) with a MiSeq Reagent Kit v3 at Shanghai Personal Biotechnology Co., Ltd. (Shanghai, China). The optimal sequencing length of the target fragment is 200 ~ 450 bp due to the short read length characteristic of MiSeq sequencing and also to ensure the sequencing quality.

### Short-chain fatty acids and lactic acid in mouse feces

Here, we identified and quantified seven short-chain fatty acids (SCFAs)—acetic acid, propionic acid, isobutyric acid, butyric acid, isovaleric acid, valeric acid, and caproic acid—in mouse fecal samples. Following the methodology outlined by a previously published report [31], we initially prepared standard curves for each of the seven SCFAs. For sample preparation, 50 mg of mouse feces were precisely weighed and placed into a sterile centrifuge tube. To this, 500 μl of sterile water containing 100 mg of glass beads was added, and the mixture was homogenized for 1 min. Following centrifugation at 4°C, 12000 rpm for 10 min, 200 μl of the supernatant was collected. To this supernatant, we added 100 μl of phosphoric acid (15%, v/v), 20 μl of an internal standard (375 μg/ml 4-methylvaleric acid), and 280 μl of ether. The mixture was then vortexed for 1 min and centrifuged again to isolate the supernatant. The resulting supernatant was subsequently analyzed using a gas chromatograph coupled with a mass spectrometer for accurate detection and quantification of the SCFAs. Gas chromatography conditions [32, 33]: the gas chromatographic (GC) analysis was conducted using a Trace 1310 gas chromatograph (Thermo Fisher Scientific, USA). The system was equipped with an Agilent HP-INNOWAX capillary column (30 m × 0.25 mm ID × 0.25 μm) and helium was employed as the carrier gas at a flow rate of 1 ml/min. Samples were injected in split mode at a ratio of 10:1, with an injection volume of 1 μl and an injector temperature maintained at 250°C. The ion source and MS transfer line temperatures were set to 300°C and 250°C, respectively. The column temperature was programmed to initiate at 90°C, then escalate to 120°C at a rate of 10°C/min, further to 150°C at 5°C/min, and finally to 250°C at 25°C/min, where it was held for 2 min. Mass spectrum conditions [32, 33]: Mass spectrometric detection of metabolites was performed on ISQ 7000 (Thermo Fisher Scientific, USA) with electron impact ionization mode. Single ion monitoring mode was used with an electron energy of 70 eV.

The supernatant derived from the stool sample in the preceding step was collected, and a commercially available Lactic Acid assay kit (Jiancheng Bioengineering Institute, Nanjing, China) was employed to ascertain the precise concentration of lactic acid present.

### Statistical analysis

The data obtained in this study were statistically analyzed using GraphPad Prism 10 (GraphPad Software, LLC, United States), and all these figures were drawn by GraphPad Prism 10. A P value less than 0.05 indicates statistical significance. Ordinary one-way ANOVA followed by Tukey's multiple comparisons test was used in the CFCS of one microorganism on the growth, biofilm production, and motility of another microorganism in a two-bacteria system; the copy number measured by qPCR; the Muc2, Occludin, Zo-1, TLR4, and IκBα expression levels; the concentrations of seven SCFAs and lactic acid in mice fecal samples. 2-way ANOVA followed by Tukey's multiple comparisons test was used in the thickness of monophyletic and symbiotic biofilms, AI-2 content, c-di-GMP content, CD growth, biofilm production, toxin protein production, and ABB indirectly inhibited CD growth by activating Raw 264.7 cells. Unpaired t-test was used in IL-6, IL-8, TNF-α, and IL-1β levels and the relative expression of TLR-4, p-65, p-IκBα, and IκBα proteins.

16S rRNA gene amplicon sequencing data were analyzed using the QIIME2 platform with slight modifications based on the official tutorial (https://docs.qiime2.org/2019.4/tutorials/). First, raw sequence data were multiplexed using the demux plugin and primers were cut using the cutadapt plugin, then sequences were quality filtered, denoised, merged and chimeras removed using the DADA2 plugin. Non-monomorphic amplicon sequence variants (ASVs) were aligned to mafft and phylogenetic models were constructed using fasttree2. Taxonomy was assigned to ASVs using the classify-sklearn naïve Bayes taxonomy classifier in the feature-classifier plugin against the SILVA release 132 databases [34]. ASV level alpha diversity indices (the observed species and Shannon index) were calculated using the ASV table in QIIME2 and visualized as box plots. ASV-level ranked abundance curves were generated to compare the richness and evenness of ASVs among samples. Beta diversity metrics were estimated using the diversity plugin with samples. Beta diversity analysis was performed to investigate the structural variation in microbial communities across samples using Bray–Curtis metrics and visualized via nonmetric multidimensional scaling (NMDS). Random forest analysis was applied to discriminate the samples from different groups using QIIME2 with default settings.

## Results

### Three bacteria engage in a mutually beneficial symbiotic relationship and are uniformly distributed within the symbiotic system

**Figure 1:**
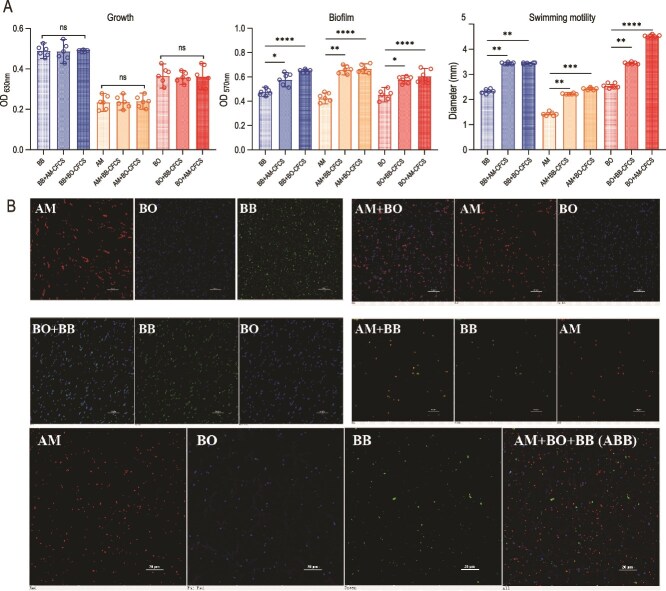
Impact of the three microorganisms on their mutual growth, biofilm formation, and cell motility, as well as the distribution of each member in the two- or three-bacteria systems. (A) Influence of the CFCS of one microorganism on the growth, biofilm production, and motility of another microorganism in a two-bacteria system. Significant differences were analyzed using ordinary one-way ANOVA followed by Tukey's multiple comparisons test, ns, *P* > .05; ^*^, *P* < .05; ^*^^*^, *P* < .01; ^*^^*^^*^, *P* < .001; ^*^^*^^*^^*^, *P* < .0001. (B) Distribution of single, dual, and triple cultures visualized using FISH probes (n = 4). Scale bar: 20 μm.

After the initial recovery, activation, and passaging, all microorganisms exhibited robust growth. Specifically, BO and BB exhibited robust activity in TSB and MRS broths, respectively. Subsequently, they were transferred to BHI broth for further cultivation. Initially, the growth kinetics of BO and BB in BHI were sluggish compared to their original growth environments. However, prolonged cultivation facilitated a gradual adaptation of BO and BB to the BHI environment, resulting in growth patterns and cycles that increasingly resembled those observed in their original media (TSB and MRS). Upon achieving growth patterns and cycles in BHI that were essentially indistinguishable from those in TSB and MRS, it can be reasonably inferred that both microorganisms have successfully acclimated to the BHI broth. After the microorganisms acclimatized to the BHI broth, it was observed that BB, AM, and BO did not interfere with each other's normal growth (Fig. 1A). Regarding biofilm formation, the CFCS of each microorganism enhanced the biofilm production of the other (Fig. 1A).

Bacterial motility is crucial for processes such as biofilm construction and the acquisition of ecological niches in the gut. We observed that following 48 h of incubation, the CFCS of each microorganism significantly enhanced the swimming performance of the other member. For instance, the swimming distance of BB increased from 2.2 mm to 3.4 mm after treatment with either AM-CFCS or BO-CFCS. The improvement in BO's swimming distance was even more pronounced, increasing nearly twofold from 2.3 mm to 4.5 mm after AM-CFCS treatment (Fig. 1A). These findings suggest that the ecological interactions among the three microorganisms exhibit commensalism in vitro, primarily evidenced by increased biofilm formation and enhanced bacterial motility.

We further labeled AM, BO, and BB using FISH probes with distinct fluorescence to observe their distribution under the co-culture system in BHI broth. We found that in the two-bacteria mixed cultures, AM and BO, BO and BB did not mix into distinct clusters, whereas AM and BB formed small, sparse clusters of mixed bacterial cells. In contrast, in the three-bacteria mixed culture (ABB), AM, BO, and BB were uniformly distributed (Fig. 1B). This feature was also evident in scanning electron microscopy (SEM) images (Fig. S1). From an ecological perspective, these three microorganisms show potential for development into mixed bacterial formulations.

### ABB develops a robust and dense symbiotic biofilm

**Figure 2:**
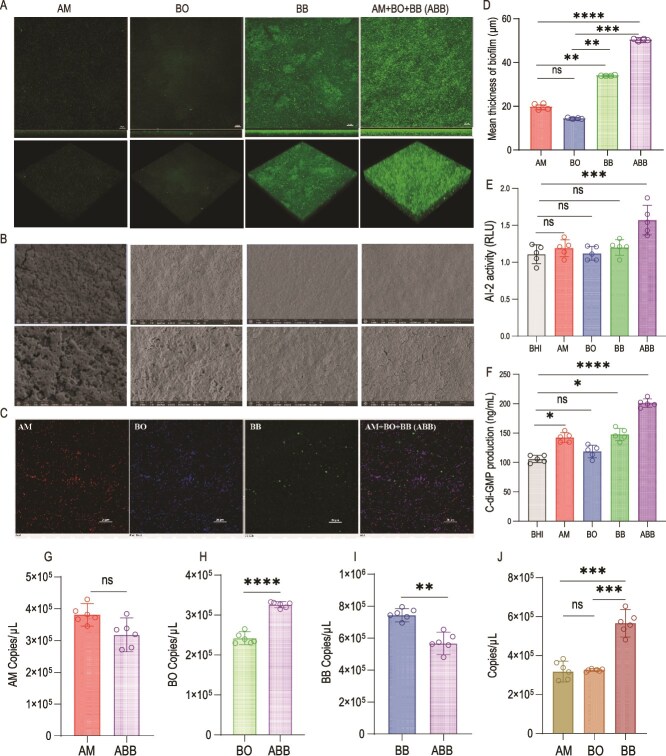
Distribution of biofilms, specifically the intrafilm members, formed in both monoculture and triculture conditions. (A) 3D images of monophyletic and symbiotic biofilms were analyzed under CLSM (n = 4). Scale in laser confocal map: 10 μm. (B) Scanning electron microscopy (SEM) was used to analyze 3D images of monophyletic and symbiotic biofilms (n = 4). Scanning electron microscope plot scale: 30 μm (top), 5 μm (bottom). (C) Distribution of AM, BO, and BB in single-bacteria biofilms and three-bacteria symbiotic biofilms labeled by FISH probes (n = 4). Scale bar: 20 μm. (D) Thickness of monophytic and symbiotic biofilms (μm) (n = 4). (E) AI-2 content in biofilm systems. (F) C-di-GMP content in biofilm systems. (G) Determination of AM copy number in monophyletic and symbiotic biofilms by qPCR. (H) Determination of BO copy number in monophyletic and symbiotic biofilms by qPCR. (I) Determination of BB copy number in monophyletic and symbiotic biofilms by qPCR. (J) Determination of AM, BO, BB copy number in symbiotic biofilms by qPCR. Significant differences were analyzed using one-way ANOVA followed by Tukey's multiple comparisons test, ns, *P > .*05; ^*^, *P < .*05; ^*^^*^, *P < .*01; ^*^^*^^*^, *P < .*001; ^*^^*^^*^^*^, *P < .*0001.

Based on the significant variation in biofilm production, confocal laser scanning microscopy (CLSM) was employed to image the structure, bacterial content, and distribution of the symbiotic biofilms formed by the mixed culture of the three microorganisms. Our findings revealed that AM and BO individually produce thin, dense monophytic biofilms lacking a distinct three-dimensional structure; BB alone generates thick, uniform, and dense monophytic biofilms with a carpet-like appearance, composed of densely arranged and continuous microaggregates, exhibiting a clear three-dimensional structure; and the symbiotic biofilms produced by the mixed cultures (ABB) are dense, carpet-like, and display a highly developed three-dimensional structure (Fig. 2A). SEM revealed that bacteria in the biofilms were encapsulated within the extracellular matrix, with bacterial cells in the ABB symbiotic biofilm being tightly arranged and intertwined to form large bacterial clusters (Fig. 2B). To investigate the distribution of AM, BO, and BB within the symbiotic biofilms, we employed FISH probes for labeling. The results demonstrated a uniform distribution of these three bacteria in the symbiotic biofilm, along with the formation of more and denser mixed bacterial cell clusters (Fig. 2C). The average thicknesses of AM, BO, and BB monophyletic biofilms were 20.00 ± 1.00 μm, 14.67 ± 0.58 μm, and 34.33 ± 0.29 μm, respectively, while the average thickness of the ABB symbiotic biofilm was 50.67 ± 1.16 μm (Fig. 2D). This indicates that the thickness of the symbiotic biofilm was significantly greater than that of the monophyletic biofilms. Additionally, we observed that the levels of AI-2 and c-di-GMP in the symbiotic biofilm system were significantly higher compared to those in the other groups (Fig. 2E, 2F).

We employed qPCR to ascertain the abundance of AM, BO, and BB within the symbiotic biofilm. Our analysis revealed that the copy number of AM remained statistically indistinguishable between the monophyletic and symbiotic biofilms (Fig. 2G). In contrast, the symbiotic biofilm exhibited a significantly elevated copy number of BO compared to the monophyletic counterpart (Fig. 2H). Conversely, the copy number of BB was observed to be reduced in the symbiotic biofilm relative to the monophyletic biofilm (Fig. 2I). Further comparative analysis of the three microorganisms within the symbiotic biofilm demonstrated that BB possessed the highest copy number among them (Fig. 2J).

### Effective inhibition of *C. Difficile* through ABB

**Figure 3:**
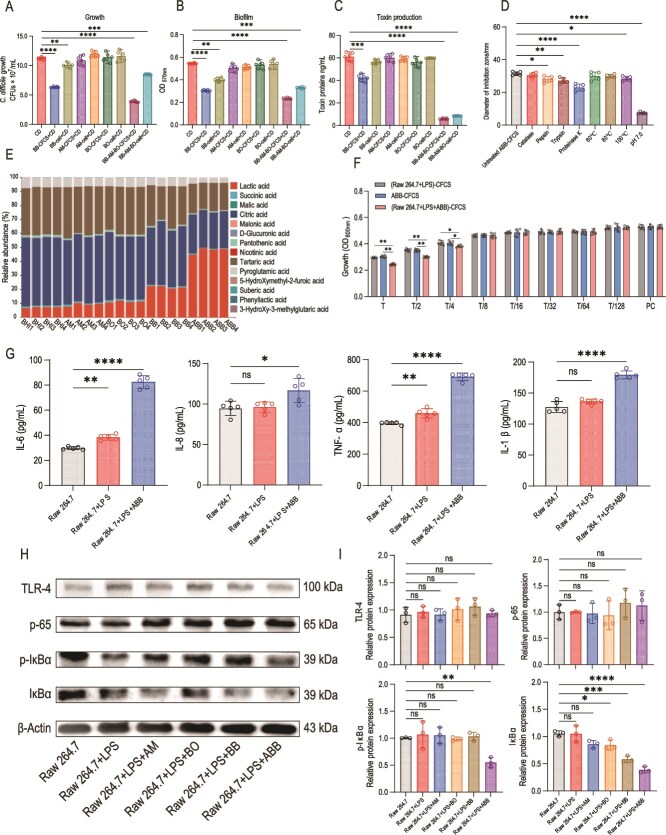
Inhibition of CD by ABB. (A) Effect of single or mixed bacterial cells (cells) or CFCS on CD growth. (B) Effect of single or mixed bacterial cells (cells) or CFCS on CD biofilm production. (C) Effect of single or mixed bacterial cells (cells) or CFCS on CD toxin protein production. (D) Diameter of the circle of inhibition of CD by ABB-CFCS under different treatments (d = mm). (E) Determination of organic acid types and content (%) in single or mixed bacterial CFCS. (F) ABB at different dilutions indirectly inhibited CD growth by activating raw 264.7 cells. The horizontal coordinate represents the dilution ratio. (G) Concentrations of IL-6, IL-8, TNF-α, and IL-1β in the supernatant after co-culture of ABB with raw 264.7 cells. (H) Activation of the TLR4/NF-κB signaling pathway in raw 264.7 cells by mono- or mixed bacterial preparations, western blot strip development plot. (I) Relative expression of TLR-4, p-65, p-IκBα, and IκBα proteins. Significant differences were analyzed in Fig. 3F using 2-way ANOVA followed by Tukey's multiple comparisons test, ^*^, *P < .*05; ^*^^*^, *P < .*01; ^*^^*^^*^, *P < .*001. Significant differences were analyzed in Fig. 3G using one-way ANOVA followed by Tukey's multiple comparisons test, ns, *P > .*05; ^*^, *P < .*05; ^*^^*^, *P < .*01; ^*^^*^^*^, *P < .*001; ^*^^*^^*^^*^, *P < .*0001. Significant differences were analyzed in Fig. 3I using the unpaired t-test. ^*^, *P < .*05; ^*^^*^, *P < .*01.

In the single-bacteria antagonizing CD test, BB-CFCS demonstrated significant inhibition of CD growth, biofilm formation, and toxin protein TcdA/B production (*P <* .05). In contrast, AM and BO exhibited no antagonistic effects against CD growth and biofilm production, whether tested using CFCS or whole cells (Fig. 3A, 3B). The mixed bacterial formulation (ABB) comprising all three microorganisms, when tested with either CFCS or cells, showed a marked inhibitory effect on CD. The production of CD toxin was significantly reduced under ABB treatment, indicating the effectiveness of the mixed bacterial formulation in inhibiting CD toxin production (Fig. 3C).

In our subsequent investigation, we conducted a comprehensive analysis of the primary antibacterial constituents secreted by ABB, ultimately ascertaining that organic acids constitute the predominant antibacterial components (Fig. 3D). Subsequently, our findings revealed that AM and BO generated minimal levels of lactic acid, while BB demonstrated a relatively substantial production of lactic acid, constituting 20% of the total organic acids. The lactic acid content in ABB-CFCS increased significantly, reaching nearly 50% of the total lactic acid content, which is more than double the amount produced by BB alone (Fig. 3E). This finding indicates that the lactic acid produced by ABB possesses the capacity to exert a direct inhibitory influence on CD within the context of in vitro experimental settings.

In addition to direct inhibition and killing of CD, we investigated whether ABB could indirectly eliminate CD by stimulating immune cells to release cytokines. To this end, we co-cultured ABB with Raw 264.7 cells and subsequently treated CD with the CFCS (Raw 264.7 + LPS + ABB). As controls, we used the CFCS from Raw 264.7 cells cultured with LPS (Raw 264.7 + LPS) and the CFCS from ABB cultured alone (ABB) (Fig. 3F). The results demonstrated that the CD biomass in the (Raw 264.7 + LPS + ABB)-CFCS treatment group was significantly reduced compared to both the (Raw 264.7 + LPS)-CFCS and ABB-CFCS treatment groups, this disparity gradually lessened with escalating dilution levels, and upon exceeding an eightfold dilution, no substantial difference remained among the three treatment groups (Fig. 3F). Furthermore, ABB was co-cultured with Raw 264.7 cells and LPS for 24 h, after which proteins from the Raw 264.7 cells were extracted for Western Blot analysis, and the cytokine levels in the supernatant were measured.

We observed that the concentrations of IL-6, IL-8, TNF-α, and IL-1β in the supernatant were markedly higher following the co-culture of ABB with Raw 264.7 cells and LPS compared to the standard culture of Raw 264.7 cells, and Raw 264.7 cells with LPS (Fig. 3G). Compared to the control group (Raw 264.7 cells and Raw 264.7 cells with LPS), the protein expression levels of NF-κB inhibitor IκBα and its phosphorylated form p-IκBα were significantly down-regulated in Raw 264.7 cells co-cultured with ABB, and this declining trend was markedly more pronounced than that observed in Raw 264.7 cells co-cultured with AM, BO, and BB (Fig. 3H, 3I). These findings suggest that ABB activates the NF-κB signaling pathway in Raw 264.7 cells. Consequently, ABB not only exerts direct cytotoxic and toxin-suppression effects on CD through its metabolites or bacterial cells but also indirectly inhibits CD by stimulating the NF-κB signaling pathway in Raw 264.7 cells.

### ABB enhances survival rates, attenuates fecal concentrations of CD toxin, decreases viable CD bacterial counts, facilitates the restoration of the intestinal barrier, and enhances the SCFA levels in mice

**Figure 4:**
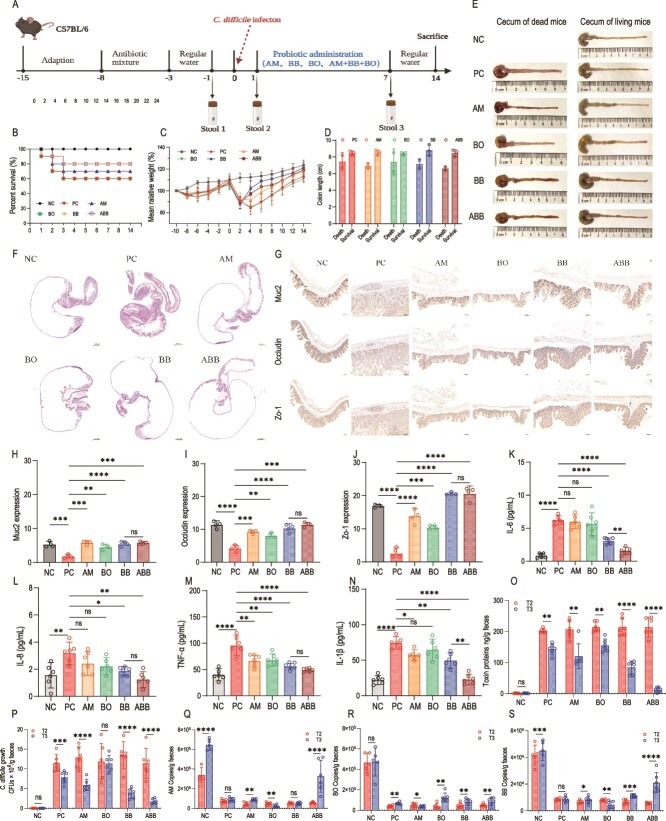
Assessment of the efficacy of the ABB formulations in a CDI mouse model. (A) Flowchart of animal experiments. (B) Final survival rate. The horizontal coordinate represents the number of days. (C) Average relative weight. The horizontal coordinate represents the number of days. (D) Colon length in dead vs. surviving mice. (E) Diagram of the cecum of dead and surviving mice (n = 4). (F) Diagram of H&E-stained sections of mouse cecum (n = 4). Scale bar: 2000 μm. (G) IHC of Muc2, Occludin, and zo-1 in mouse cecum (n = 4). Scale bar: 100 μm. (H) Muc2 expression level (n = 4). (I) Occludin expression levels (n = 4). (J) Zo-1 expression level (n = 4). (K) IL-6 levels in colonic tissue at T3 (n = 6). (L) IL-8 levels in colonic tissue at T3 (n = 6). (M) TNF-α levels in colonic tissue at T3 (n = 6). (N) IL-1β levels in colonic tissue at T3 (n = 6). (O) Changes in CD toxin levels in mouse feces before and after treatment. (P) Changes in CD number in mouse feces before and after treatment. (Q) Determination of AM copy number in mouse feces before and after treatment by qPCR. (R) Determination of BO copy number in mouse feces before and after treatment by qPCR. (S) Determination of BB copy number in mouse feces before and after treatment by qPCR. Significant differences were analyzed in Fig. 4H, 4I, 4J, 4K, 4L, 4M, 4N using one-way ANOVA followed by Tukey's multiple comparisons test, ns, *P > .*05; ^*^, *P < .*05; ^*^^*^, *P < .*01; ^*^^*^^*^, *P < .*001; ^*^^*^^*^^*^, *P < .*0001. Significant differences were analyzed in Fig. 4O, 4P, 4Q, 4R, 4S using the unpaired t-test, ns, *P > .*05; ^*^, *P < .*05; ^*^^*^, *P < .*01; ^*^^*^^*^, *P < .*001; ^*^^*^^*^^*^, *P < .*0001.

Microecological formulations intervening at the first sign of clinical symptoms (weight loss and loose stools) in mice. The final survival rates were as follows: NC (100%) > ABB (80%) = BB (80%) > AM (70%) > BO (60%) = PC (60%) (Fig. 4A, 4B). Treatment with BO alone did not improve mouse survival, whereas ABB and BB treatments were effective. The weight of the mice decreased significantly within 2 d post-infection. Mice treated with ABB and BB began to regain weight by day 4, while those treated with AM or BO alone continued to lose weight for 2 d after administration before beginning to recover (Fig. 4C). Except for the NC group, death occurred in the remaining groups. We collected cecum and colon tissues from both deceased and surviving mice at the endpoint of the experiment for measurements. The results showed that the infection significantly shortened the length of the colon in mice (Fig. 4D, 4E). Additionally, H&E-stained images of the cecum sections revealed severe damage to the intestinal villi and the epithelial cell layer in infected mice, while the morphology of the cecum sections from the surviving mice closely resembled that of the NC group (Fig. 4F).

By immunohistochemical analysis, we observed that the cecum morphology of mice in the PC group exhibited significant folding, and the protein expression levels of Muc2, Occludin, and ZO-1 were markedly reduced. In contrast, mice in the treatment group showed a diametrically opposite trend, with the expression levels of Muc2, Occludin, and ZO-1 being significantly elevated following ABB and BB intervention, restoring them to levels comparable to or exceeding those of the NC group (Fig. 4G-J). We observed that following the completion of the treatment, the levels of IL-6, IL-8, TNF-α, and IL-1β in the colon tissues of mice in the ABB and BB groups were significantly decreased compared to the PC group. The down-regulation of IL-6 and IL-1β levels was more pronounced in the ABB group compared to the BB group (Fig. 4K-N). Studies have demonstrated that both AM and BB can exert anti-inflammatory effects by inhibiting the NF-κB signaling pathway and reducing the production of inflammatory factors such as IL-6 and IL-8 [35, 36], which is consistent with our findings. Upon treatment conclusion, the levels of these cytokines in the ABB group approached those of the NC group.

The CD toxin protein content and viable bacteria counts in feces serve as crucial indicators for evaluating treatment efficacy and the rates of recurrent infection. Our findings revealed a decline in both the CD toxin protein content and the viable bacterial counts across all groups at T3, including the PC group (Fig. 4O, 4P, Fig. S2). When using the PC group at T3 as a benchmark, we observed a significant reduction in these parameters in the AM, BB, and ABB groups (Fig. 4O). Among the groups, the ABB group exhibited a significantly lower concentration of toxin protein and CD viable bacterial counts compared to the BB group, with levels approaching those observed in the NC group (Fig. S2). Consequently, in terms of substantial efficacy and minimizing the recurrent infection rate, the ABB treatment emerged as the most effective. The quantities of AM, BO, and BB in mouse feces were determined through qPCR analysis. Our results indicated that at T3, the copy numbers for each individual bacterial group were elevated compared to the pre-infection levels (AM, BO, BB). The copy numbers of AM and BB in the ABB group were markedly higher than those in all other groups, except for the NC group (Fig. 4Q, 4R, 4S, Fig. S2). The copy numbers of AM and BB in the ABB group were substantially higher than those observed in the BB group. These findings suggest that the ABB treatment effectively enhances the populations of AM and BB within the mouse gut.

**Figure 5:**
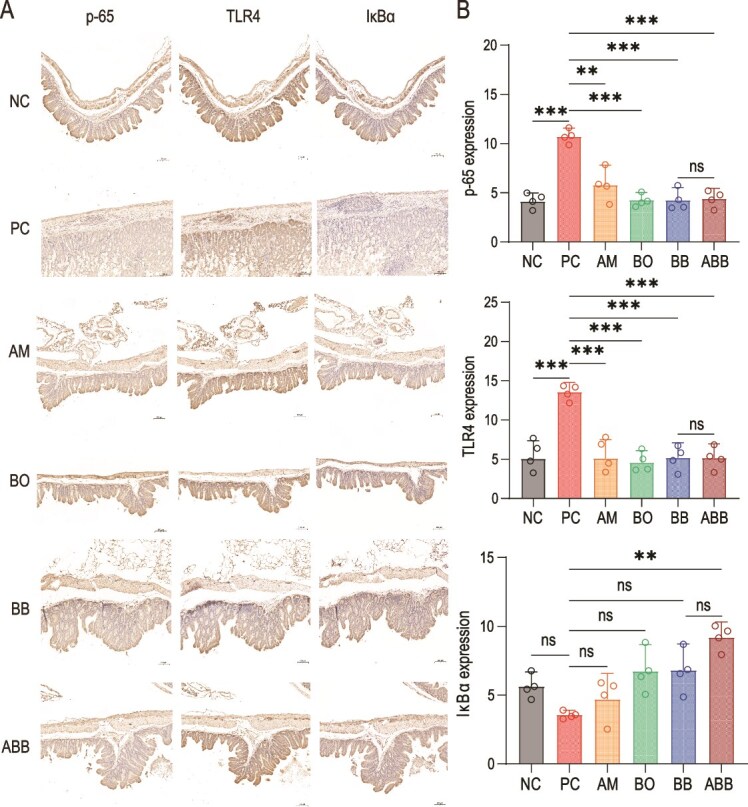
Immunohistochemistry (IHC) of p-65, TLR4, and IκBα in the cecum of mice in each group. (A) Immunohistochemical pictures of cecum p-65, TLR4, and IκBα. (B) p-65, TLR4, and IκBα expression levels (n = 4). Scale bar: 100 μm. Significant differences were analyzed using ordinary one-way ANOVA followed by Tukey's multiple comparisons test, ns, *P > .*05, ^*^^*^, *P < .*01; ^*^^*^^*^, *P < .*001; ^*^^*^^*^^*^, *P < .*0001.

Compared with the NC group, the protein expression levels of p-65 and TLR4 were markedly elevated in the PC group, while the expression of the NF-κB inhibitor IκBα was concurrently downregulated (Fig. 5A). This suggests that the activation of the NF-κB signaling pathway in the PC group could result in an increased release of inflammatory factors, thereby exacerbating CD-induced enteritis. Compared with the PC group, the protein expression levels of p-65, TLR4, and IκBα were restored to levels comparable to those of the NC group in both the single- and triple-bacteria administration groups (Fig. 5B). This suggests that the administration of the ABB formulation can mitigate the intestinal inflammatory response induced by the activation of the NF-κB pathway due to CDI.

**Figure 6:**
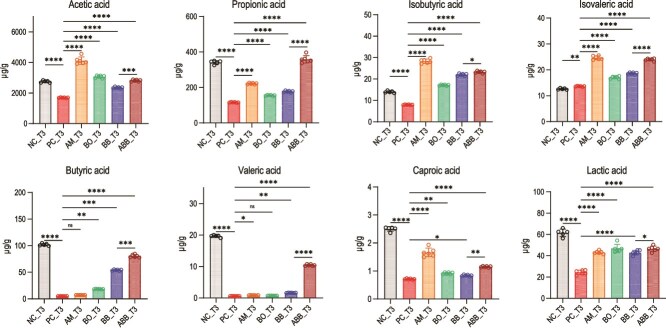
Concentrations of seven SCFAs and lactic acid in mice fecal samples. Significant differences were analyzed using ordinary one-way ANOVA followed by Tukey's multiple comparisons test, ns, *P > .*05; ^*^, *P < .*05; ^*^^*^, *P < .*01; ^*^^*^^*^, *P < .*001; ^*^^*^^*^^*^, *P < .*0001.

In our investigation, we conducted a comprehensive analysis of the SCFAs and lactic acid content in the fecal samples of mice at T3. Our results revealed a marked increase in the concentrations of both SCFAs and lactic acid at the conclusion of the treatment period, relative to the concurrent PC group (Fig. 6). Specifically, within the SCFA profile, the levels of acetic acid, propionic acid, isobutyric acid, butyric acid, valeric acid, and caproic acid were substantially elevated in the ABB group when compared to the BB group. Concurrently, the lactic acid concentration in the ABB group was also elevated compared to that in the BB group (Fig. 6).

Based on the data pertaining to the final survival rate and body weight index of the mice, both ABB and BB demonstrated substantial therapeutic efficacy. However, a comparative analysis of the colonic cytokine levels, fecal CD toxin content, CD viable counts, SCFAs content, and lactic acid level revealed that the therapeutic effects exerted by ABB were markedly superior to those of BB.

### ABB facilitates the reconstitution of gut microbiota in mice

**Figure 7:**
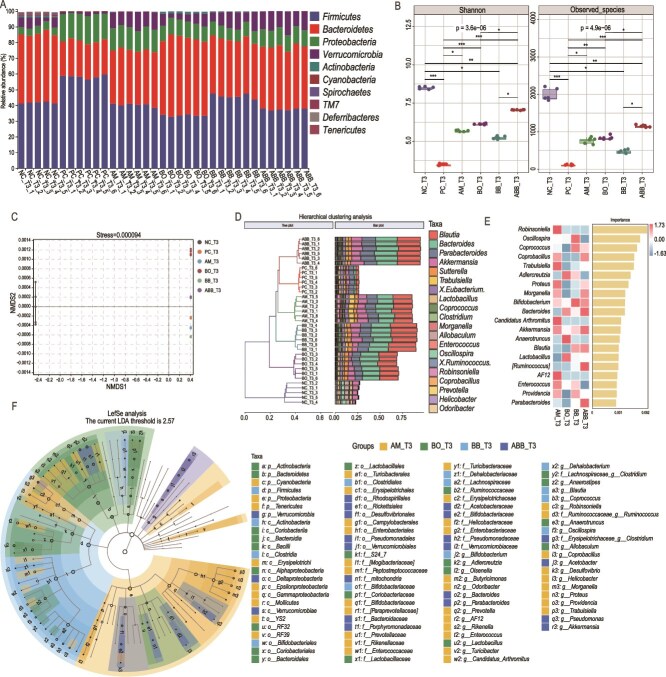
Analysis of the gut microbiota at the T3. (A) The top 10 family-level bacteria in terms of relative abundance. (B) Alpha diversity levels. The p-value produced by the Kruskal–Wallis test. (C) Non-metric multidimensional scaling analysis (NMDS) of the Bray-Curtis distance matrix. (D) Hierarchical clustering analysis. The bray-Curtis distance matrix was analyzed for clustering using the UPGMA algorithm. Top 20 genus-level bacteria in terms of abundance. (E) Random forest. Absolute abundance table of the top 20 genus-level members in terms of importance, with the horizontal coordinates of the bar graphs being the importance of the bacteria to the classifier model scored as the importance of the bacteria to the model, with bacteria decreasing in importance to the model from the top to the bottom; it can be assumed that these top-ranked bacteria in terms of importance are the marker bacteria for the differences between the groups. (F) LEfSe analysis of keystone member. The taxonomic branching diagram shows the taxonomic rank relationships of the major taxonomic units from phylum to genus (inner to outer ring) in the sample community.

We analyzed the gut microbiota of mice at T3. The abundance of Bacteroidetes, Verrucomicrobia, and Actinobacteria was increased in the gut of treated mice compared to the PC group, reaching levels similar to those of the NC group (Fig. 7A). Regarding microbial alpha diversity, the treated mice exhibited higher Shannon and Observed_species indices than the PC group. Specifically, the levels of these two indicators were higher in the ABB group compared to the BB group (Fig. 7B). Additionally, the bacterial population distribution in each treatment group showed significant interspecies differences at T3 (Fig. 7C). Analysis of the top 20 genus-level bacteria by abundance revealed that Blautia, Bacteroides, Parabacteroides, Akkermansia, and Bifidobacterium collectively accounted for nearly 75% of the post-treatment groups (Fig. 7D). Using Random Forest analysis, we identified Bifidobacterium, Bacteroides, and Akkermansia as the top-ranked genera with high absolute abundance (Fig. 7E). Enterococcus, another top-ranked genus, exhibited a significant decrease in abundance only in the ABB group while maintaining high levels in the AM, BO, and BB groups (Fig. 7E, 7F).

## Discussion

Intestinal infections caused by CD represent a form of bacterial enteritis, characterized by significant disruptions in gut ecology. These disruptions manifest as substantial alterations in metabolites, such as SCFAs, bile acids, and fermentable amino acids, as well as changes in the composition of the microbial community [1]. Although the use of traditional antibiotics like VAN remains the primary treatment for CDI, this approach can exacerbate host gut ecological imbalances and infections [37]. The antibiotic environment enables antibiotic-resistant CD strains and their endospore to germinate, re-colonize, and produce toxins, simultaneously, this environment fosters the excessive proliferation of antibiotic-resistant members in the Enterococcaceae and Enterobacteriaceae within the gut, ultimately leading to severe co-infections [4, 6, 16]. FMT has demonstrated promising outcomes for refractory rCDI; however, numerous uncertainties remain, such as the potential presence of harmful bacteria and virulence factors in donor feces, which restrict its application in pediatric CDI patients or immunocompromised individuals [1]. Moreover, FMT involves a large-scale alteration of the host’s gut microbiome, and the long-term effects on the host’s immune system and major metabolic processes are yet to be fully understood. However, it is important to highlight that FMT has shown a high cure rate and an extremely low recurrence rate in rCDI, indicating that artificial interventions in the gut microbiota hold significant potential for the management of CDI. A recent study by Furuichi et al. was also motivated by the FMT, where a select group of microorganisms is artificially combined to modulate the microbial structure, creating a bacterial blend capable of containing infections and alleviating inflammation by depriving Enterobacteriaceae of their intestinal ecological niche [4]. Therefore, from the perspective of precision therapy, the development of personalized microecological agents holds significant potential for application in intestinal infections and inflammation modulation. Recent therapeutic advancements for rCDI have seen the emergence of rationally designed microbial consortia, VE303 (developed by Menon's team) and SER-109 (pioneered by Feuerstadt's group), both demonstrating statistically significant efficacy in randomized controlled trials [10, 11]. However, it is crucial to acknowledge that the long-term impact of these microbial formulations, given their vast numbers and intricate composition, on the host’s inherent gut microbiota, remains uncertain. There is a potential for interference with the native inhabitants of the host’s intestinal tract, and their complex ecological interactions necessitate further elucidation. Moreover, the presence or absence of individual bacteria within these formulations may not significantly influence the overall efficacy, suggesting that unnecessary components should be eliminated to mitigate potential risks. From the perspective of minimizing the complexity of microcosmic formulations while ensuring their effectiveness, the development of formulations with the smallest possible size is a key focus for future research endeavors. These formulations, characterized by their relatively simple composition, enable targeted adjustments to the host's gut microbiota, ensuring the desired therapeutic outcomes while minimizing interference with native microbiota and avoiding potential adverse effects. Probiotics, as a prime example of live microecological preparations, align well with this concept.

In recent years, there has been a growing consensus on developing novel therapies for CDI by focusing on the ecological regulation of gut microbiota. This approach primarily encompasses the regulation of gut nutrients and the modulation of gut microbial community structure. Gut nutrient regulation involves the management of bile acids (both primary and secondary) and Stickland amino acids (such as ornithine, proline, and arginine). The regulation of gut microbial community structure aims to enhance the presence of beneficial bacteria while reducing the abundance of harmful ones [5]. The recurrence rate of CDI during antibiotic treatment can reach up to 25%, largely due to the collaborative efforts of certain gut bacteria that protect CD from antibiotic-induced death [2]. Among these, Enterococcus has been fully established as the primary accomplice. Smith et al. demonstrated that Enterococcus alters the intestinal amino acid environment by providing substantial amounts of leucine and ornithine, which facilitates CD colonization in the gut [6]. Similarly, Engevik et al. reported that *F. nucleatum* can form a denser biofilm with CD by co-aggregating with it, thereby enhancing CD colonization in the mucosal layer [8]. Our group also observed in a previous study that *Bacteroides thetaiotaomicron* can form a thicker symbiotic biofilm with CD in the VAN environment, which increases the survival rate of CD [16]. Of course, in addition to these CD allies, there are several hostile microorganisms in the host gut. The vast majority of these beneficial bacteria, such as Bifidobacterium and *A. muciniphila*, are highly sensitive to antibiotics and thus experience a significant decline during antibiotic interventions [9]. From the perspective of microbiota homeostasis, the lack of beneficial members results in an imbalance in the gut ecosystem. This adverse condition is challenging to completely restore through medication alone, as the absence of beneficial bacteria can become a persistent state. From a “what goes around comes around” perspective, human intervention to replenish these missing beneficial members could enhance the efficacy of conventional treatments and mitigate the risk of reinfection. Our prior research revealed that the CDI process is characterized by a significant reduction in the abundance of Akkermansia, Bacteroides, and Bifidobacterium, with AM, BO, and BB being identified as key bacteria. Therefore, we hypothesized that the relationship among these three microorganisms is likely to be synergistic and mutualistic, suggesting that a tripartite mixed bacterial formulation derived from them could potentially possess efficacy for treating CDI. In vitro experiments have demonstrated that AM, BO, and BB exhibit mutually beneficial symbiosis and synergistic support, exemplified by the formation of more robust symbiotic biofilms. Symbiotic biofilms facilitate the robust colonization of intramembrane bacteria in the intestinal tract while simultaneously mitigating or preventing the lethal effects of antibiotics on these bacteria, thereby substantially enhancing their survival rates [38]. This dual characteristic not only facilitates the proliferation and potential infection of pathogenic bacteria (such as CD or *E. faecalis*) within the human gut but also safeguards beneficial members from antibiotic-induced death, thereby enhancing their colonization and enables them to more effectively exert their probiotic functions. In our prior research, we demonstrated the efficacy of combining BB, VAN, and metronidazole in the treatment of pCDI in mice [29]. The direct lethal effect of antibiotics on BB persists, even when they are administered several hours before the antibiotic treatment. Considering the protective role that biofilms play for microorganisms, the combination of ABB with conventional antibiotics may yield a synergistic therapeutic effect, enhancing the overall efficacy of the treatment. This potential is attributed to ABB’s capability to develop more robust symbiotic biofilms, which not only facilitate the colonization of the bacteria but also mitigate the direct lethal effects of antibiotics on these bacteria. Hence, the therapeutic strategy of merging ABB with antibiotics warrants further investigation and validation.

Microorganisms engage in diverse interactions, both direct and indirect [39]. Our study revealed that ABB, a composite bacterial formulation comprising AM, BO, and BB, effectively increased lactic acid production, which directly inhibited the growth of CD. Lactic acid is known to create an unfavorable environment for harmful microorganisms by modulating intestinal pH [26]. Simultaneously, microorganisms can indirectly influence other members by enhancing host immunity. The ABB has been shown to indirectly suppress CD by activating the NF-κB signaling pathway in Raw 264.7 cells, leading to the secretion of substantial amounts of L-6, IL-8, TNF-α, and IL-1β. In our investigation, we ascertained that both ABB and BB were efficacious in enhancing survival rates and augmenting body mass in CDI murine models. Nevertheless, ABB exhibited a more comprehensive array of beneficial effects when compared to BB. These included a pronounced down-regulation of inflammation-associated cytokine secretion within colonic tissues, a reduction in CD toxin protein levels, and a decrease in viable CD counts. Additionally, ABB facilitated an elevation in the concentrations of SCFAs, with a particular emphasis on propionic, butyric, and valeric acids, alongside an increase in the populations of AM and BB within the gastrointestinal tract. Furthermore, ABB was observed to expedite the restoration of the intestinal barrier in infected mice and ameliorate intestinal inflammation through the downregulation of key protein expression levels, such as p-65, TLR4, and IκBα, which are integral to the NF-κB signaling pathway. In the context of gut ecological regulation, our findings revealed that the alpha diversity of microbiota in mice subjected to ABB intervention was markedly higher compared to those in the AM, BO, and BB treatment groups. The relative abundance of Blautia, Bacteroides, Parabacteroides, Akkermansia, and Bifidobacterium was elevated. These results indicate that ABB effectively restored the homeostasis of the gut microbiota in mice and replenished beneficial members such as Bacteroides, Akkermansia, and Bifidobacterium, which were depleted during the infection. This restoration sets the stage for sustained and effective combat against CD and its associated bacteria (e.g., Enterococcus) in subsequent stages. The abundance of Enterococcus decreased sharply in response to the ABB intervention, while it remained at a certain level in the other treatment groups. The absence of Enterococcus can deprive CD of a key ally, thereby reducing the likelihood of future recurrent infections. Although the ABB and BB interventions had similar effects on mouse survival, the ABB intervention resulted in significantly higher microbial diversity and a marked reduction in Enterococcus abundance in the intestines of recovered mice. Collectively, the mutualistic and symbiotic relationship between AM, BO, and BB leads to an effective ABB formulation that can alleviate intestinal inflammation, promote the reconstruction of the gut microbiota in CDI mice, and increase the abundance of Bacteroides, Akkermansia, and Bifidobacterium. This mixed-bacteria formulation holds promise as a novel therapeutic option for clinical CDI treatment. However, it is imperative to recognize the inherent limitations of this study. Specifically, the in vitro biological parameters of the ABB formulation and its demonstrated effects in mouse models may not fully reflect its true clinical efficacy, owing to the significantly more complex intestinal ecology present in the human body. Further investigation is warranted to ascertain any alterations in the ecological interactions among the strains within the ABB formulation when introduced to the human gut. Concurrently, the actual clinical efficacy of the formulation necessitates validation through experiments conducted on a sufficient scale.

## Supplementary Material

Supplementary_materials_wraf086

## Data Availability

All sequencing data are available at the Sequence Read Archive (SRA) of NCBI (PRJNA1183614).

## References

[1] Smits WK, Lyras D, Lacy DB. et al. Clostridium difficile infection. *Nat Rev Dis Primers* 2016;2:16020. 27158839 10.1038/nrdp.2016.20 PMC5453186

[2] Yang J, Yang H. Non-antibiotic therapy for Clostridioides difficile infection: a review. *Crit Rev Clin Lab Sci* 2019;56:493–509. 10.1080/10408363.2019.1648377 31411909

[3] Sehgal K, Cifu AS, Khanna S. Treatment of Clostridioides difficile infection. *JAMA* 2022;328:881–2. 10.1001/jama.2022.12251 35939317

[4] Furuichi M, Kawaguchi T, Pust M-M. et al. Commensal consortia decolonize Enterobacteriaceae via ecological control. *Nature* 2024;633:878–86. 10.1038/s41586-024-07960-6 39294375 PMC11424487

[5] Pruss KM, JLC S. Difficile exploits a host metabolite produced during toxin-mediated disease. *Nature* 2021;593:261–5. 10.1038/s41586-021-03502-6 33911281 PMC9067157

[6] Smith AB, Jenior ML, Keenan O. et al. Enterococci enhance Clostridioides difficile pathogenesis. *Nature* 2022;611:780–6. 10.1038/s41586-022-05438-x 36385534 PMC9691601

[7] Fletcher JR, Pike CM, Parsons RJ. et al. Clostridioides difficile exploits toxin-mediated inflammation to alter the host nutritional landscape and exclude competitors from the gut microbiota. *Nat Commun* 2021;12:462. 33469019 10.1038/s41467-020-20746-4 PMC7815924

[8] Engevik MA, Danhof HA, Auchtung J. et al. Fusobacteriumnucleatum adheres to Clostridioides difficile via the RadD adhesin to enhance biofilm formation in intestinal mucus. *Gastroenterology* 2021;160:1301–14. 10.1053/j.gastro.2020.11.034 33227279 PMC7956072

[9] Yang J, Meng L, Li Y. et al. Strategies for applying probiotics in the antibiotic management of Clostridioides difficile infection. *Food Funct* 2023;14:8711–33. 10.1039/D3FO02110F 37725066

[10] Menon R, Bhattarai SK, Crossette E. et al. Multi-omic profiling a defined bacterial consortium for treatment of recurrent Clostridioides difficile infection. *Nat Med* 2025;31:223–34. 10.1038/s41591-024-03337-4 39747680

[11] Feuerstadt P, Louie TJ, Lashner B. et al. SER-109, an oral microbiome therapy for recurrent Clostridioides difficile infection. *N Engl J Med* 2022;386:220–9. 10.1056/NEJMoa2106516 35045228

[12] Battaglioli EJ, Hale VL, Chen J. et al. Clostridioides difficile uses amino acids associated with gut microbial dysbiosis in a subset of patients with diarrhea. *Sci Transl Med* 2018;10:eaam7019. 10.1126/scitranslmed.aam7019 30355801 PMC6537101

[13] Lesniak NA, Schubert AM, Flynn KJ. et al. The gut bacterial community potentiates Clostridioides difficile infection severity. *MBio* 2022;13:e0118322. 10.1128/mbio.01183-22 35856563 PMC9426473

[14] Dong Q, Harper S, McSpadden E. et al. Protection against Clostridioides difficile disease by a naturally avirulent strain. *Cell Host Microbe* 2025;33:59–70. 10.1016/j.chom.2024.11.003 39610252 PMC11731898

[15] Zhong S, Yang J, Huang H. Efficacy assessment of the co-administration of vancomycin and metronidazole in Clostridioides difficile-infected mice based on changes in intestinal ecology. *J Microbiol Biotechnol* 2024;34:828–37. 10.4014/jmb.2312.12034 38668685 PMC11091681

[16] Yang J, Rui W, Zhong S. et al. Symbiotic biofilms formed by Clostridioides difficile and bacteroides thetaiotaomicron in the presence of vancomycin. *Gut Microbes* 2024;16:2390133. 10.1080/19490976.2024.2390133 39132815 PMC11321409

[17] Swidsinski A, Dörffel Y, Loening-Baucke V. et al. Acute appendicitis is characterised by local invasion with fusobacterium nucleatum/necrophorum. *Gut* 2011;60:34–40. 10.1136/gut.2009.191320 19926616

[18] Berry D, Stecher B, Schintlmeister A. et al. Host-compound foraging by intestinal microbiota revealed by single-cell stable isotope probing. *Proc Natl Acad Sci USA* 2013;110:4720–5. 10.1073/pnas.1219247110 23487774 PMC3607026

[19] Berry D, Schwab C, Milinovich G. et al. Phylotype-level 16S rRNA analysis reveals new bacterial indicators of health state in acute murine colitis. *ISME J* 2012;6:2091–106. 10.1038/ismej.2012.39 22572638 PMC3475367

[20] Lin LY, Song J, Li J. et al. Imaging the in vivo growth patterns of bacteria in human gut microbiota. *Gut Microbes* 2021;13:1960134. 10.1080/19490976.2021.1960134 34428120 PMC8386752

[21] Dinoto A, Suksomcheep A, Ishizuka S. et al. Modulation of rat cecal microbiota by administration of raffinose and encapsulated. *Appl Environ Microbiol* 2006;72:784–92. 10.1128/AEM.72.1.784-792.2006 16391119 PMC1352276

[22] Schneeberger M, Everard A, Gómez-Valadés AG. et al. Akkermansia muciniphila inversely correlates with the onset of inflammation, altered adipose tissue metabolism and metabolic disorders during obesity in mice. *Sci Rep* 2015;5:16643. 10.1038/srep16643 26563823 PMC4643218

[23] Kato K, Odamaki T, Mitsuyama E. et al. Age-related changes in the composition of gut species. *Curr Microbiol* 2017;74:987–95. 10.1007/s00284-017-1272-4 28593350 PMC5486783

[24] Matsuki T, Watanabe K, Fujimoto J. et al. Quantitative PCR with 16S rRNA-gene-targeted species-specific primers for analysis of human intestinal bifidobacteria. *Appl Environ Microb* 2004;70:167–73. 10.1128/AEM.70.1.167-173.2004 PMC321263 14711639

[25] Ishikawa E, Matsuki T, Kubota H. et al. Ethnic diversity of gut microbiota: species characterization of Bacteroides fragilis group and genus Bifidobacterium in healthy Belgian adults, and comparison with data from Japanese subjects. *J Biosci Bioeng* 2013;116:265–70. 10.1016/j.jbiosc.2013.02.010 23522670

[26] Rui W, Gu C, Zhang H. et al. Antagonistic activity of selenium-enriched Bifidobacterium breve against Clostridioides difficile. *Appl Microbiol Biotechnol* 2022;106:6181–94. 10.1007/s00253-022-12124-5 35962282

[27] Yang J, Yang H. Effect of Bifidobacterium breve in combination with different antibiotics on Clostridium difficile. *Front Microbiol* 2018;9:2953. 10.3389/fmicb.2018.02953 30564210 PMC6288195

[28] Park SY, Ji GE, Ko YT. et al. Potentiation of hydrogen peroxide, nitric oxide, and cytokine production in RAW 264.7 macrophage cells exposed to human and commercial isolates of Bifidobacterium. *Int J Food Microbiol* 1999;46:231–41. 10.1016/S0168-1605(98)00197-4 10100903

[29] Yang J, Meng L, Yang H. Therapeutic effects of Bifidobacterium breve YH68 in combination with vancomycin and metronidazole in a primary Clostridioides difficile-infected mouse model. *Microbiol Spectr* 2022;10:e0067222. 10.1128/spectrum.01451-22 35311540 PMC9045379

[30] Yang JP, Yang H. Evaluation of the therapeutic effect and dose-effect of Bifidobacterium breve on the primary Clostridioides difficile infected mice. *Appl Microbiol Biotechnol* 2021;105:9243–60. 10.1007/s00253-021-11668-2 34751791

[31] Han X, Guo J, You Y. et al. A fast and accurate way to determine short chain fatty acids in mouse feces based on GC-MS. *J Chromatogr B Analyt Technol Biomed Life Sci* 2018;1099:73–82. 10.1016/j.jchromb.2018.09.013 30243116

[32] Zhang S, Wang H, Zhu M-J. A sensitive GC/MS detection method for analyzing microbial metabolites short chain fatty acids in fecal and serum samples. *Talanta* 2019;196:249–54. 10.1016/j.talanta.2018.12.049 30683360

[33] Hsu Y-L, Chen C-C, Lin Y-T. et al. Evaluation and optimization of sample handling methods for quantification of short-chain fatty acids in human fecal samples by GC-MS. *J Proteome Res* 2019;18:1948–57. 10.1021/acs.jproteome.8b00536 30895795

[34] Bokulich NA, Kaehler BD, Rideout JR. et al. Optimizing taxonomic classification of marker-gene amplicon sequences with QIIME 2' s q2-feature-classifier plugin. *Microbiome* 2018;6:90. 10.1186/s40168-018-0470-z 29773078 PMC5956843

[35] Tomosada Y, Villena J, Murata K. et al. Immunoregulatory effect of Bifidobacteria strains in porcine intestinal epithelial cells through modulation of ubiquitin-editing enzyme A20 expression. *PLoS One* 2013;8:e59259. 10.1371/journal.pone.0059259 23555642 PMC3608626

[36] Shi J, Wang F, Tang L. et al. Akkermansia muciniphila attenuates LPS-induced acute kidney injury by inhibiting TLR4/NF-κB pathway. *FEMS Microbiol Lett* 2022;369:fnac103. 36368696 10.1093/femsle/fnac103

[37] Guh AY, Kutty PK. Clostridioides difficile infection. *Ann Intern Med* 2018;169:ITC49-ITC64. 30285209 10.7326/AITC201810020 PMC6524133

[38] Zhong S, Yang J, Huang H. The role of single and mixed biofilms in Clostridioides difficile infection and strategies for prevention and inhibition. *Crit Rev Microbiol* 2024;50:285–99. 10.1080/1040841X.2023.2189950 36939635

[39] Yang J, Yang H, Li Y. The triple interactions between gut microbiota, mycobiota and host immunity. *Crit Rev Food Sci Nutr* 2023;63:11604–24. 10.1080/10408398.2022.2094888 35776086

