# Supplementary material for: Bifidobacterium breve synergizes with Akkermansia muciniphila and Bacteroides ovatus to antagonize Clostridioides difficile
Source: ISME J. 2025 Apr 30;19(1):wraf086. doi: 10.1093/ismejo/wraf086 (PMC12089032; doi:10.1093/ismejo/wraf086)
Supplement: Supplementary_materials_wraf086 [file supplementary_materials_wraf086.pdf]

Supplementary materials

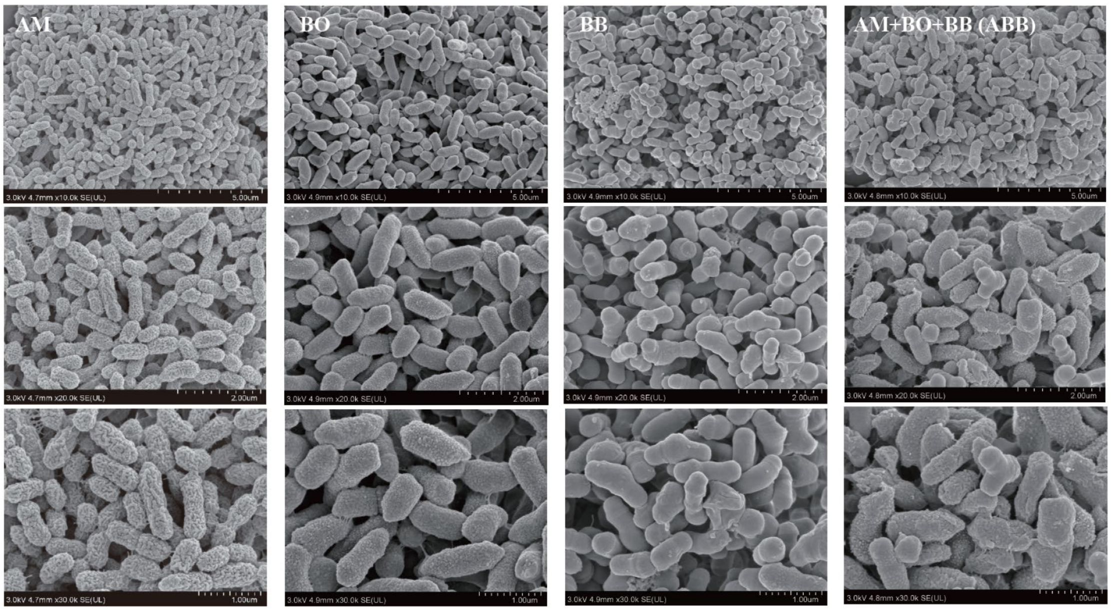

Figure S1. Scanning electron microscopy (SEM) images of single and triple bacteria in the three-bacteria systems (n=4). Scale bars: 5 μm (top), 2 μm (middle), 1 μm (bottom).

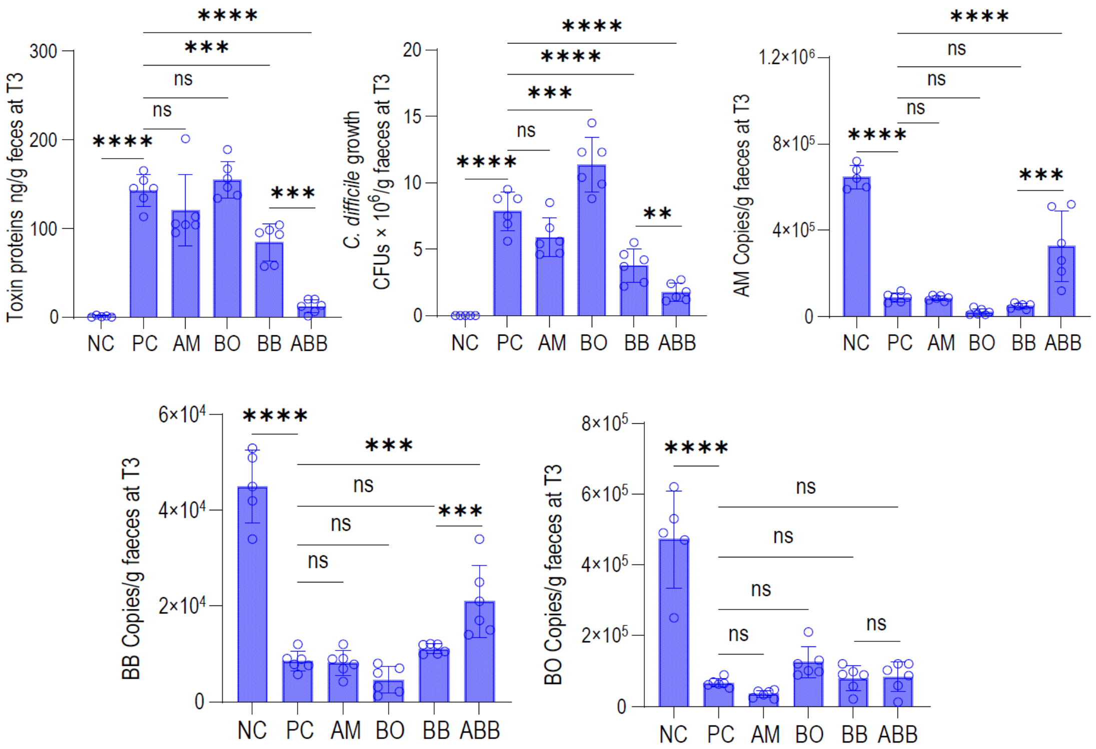

Figure S2. CD toxin levels, CD number, AM copy number, BO copy number, BB copy number at the T3. Significant differences were using one-way ANOVA followed by Tukey's multiple comparisons test, ns, $P>0.05$; *, $P<0.05$; **, $P<0.01$; ***, $P<0.001$; ****, $P<0.0001$.

Table S1 Target genes in AM, BO, and BB

Strain	Target gene sequence (5 '-3')	length
AM	CCTTGCGGTTGGCTTCAGATACTTCGGGTGCGACCGGCTTCCATGATGTGACGGGCGGTGTGTACAAGACCCGGGAACGTATTCACGGCGCCGTAGCTGATGCGCCATTACTAGCGATTCCGGCTTCGTGTAGGCGGGTTGCAGCCTACAGTCCGAACTGGGCCCAGTTTTTAGGATTTCCTCCGCCTCGCGGCTTCGGCCCCCTCTGTACTGGGCATTGTAGTACGTGTGCAGCCCTGGGCATAAGGGCCATACTGACCTGACGTCGTCCCCACCTTCCTCCCAGTTGATCTGGGCAGTCTCGCCAGAGTCCCCACCTTCACGTGCTG	329bp
BB	CCGGATGCTCCATCACACCGCATGGTGTGTTGGGAAAGCCTTTGCGGCATGGGATGGGGTCGCGTCCTATCAGCTTGATGGCGGGGTAACGGCCCACCATGGCTTCGACGGGTAGCCGGCCTGAGAGGGCGACCGGCCACATTGGGACTGAGATACGGCCCAGACTCCTACGGGAGGCAGCAGTGGGGAATATTGCACAATGGGCGCAAGCCTGATGCAGCGACGCCGCGTGAGGGATGGAGGCCTTCGGGTTGTAAACCTCTTTTATCAGGGAGCAAGGCACTTTGT	288bp
BO	CACAACTGACTTAACAATCCACCTACGCTCCCTTTAAACCCAATAAATCCGGATAACGCTCGGATCCTCCGTATTACCGCGGCTGCTGGCACGGAGTTAGCCGATCCTTATTCATATGGTACATACAAAATTCCACACGTGGAAAACTTTATTCCCATATAAAAGAAGTTTACGACCCATAGAGCCTTCATCCTTCACGCTACTTGGCTGGTTCAGGCTCTCGCCCATTGACCAATATTCCTCACTGCTGCCTCCCGTAGGAGTTTGGACCGTGTCTCAGTTCCAATGTGGGGGACCTTCCTCTCAGAACCCCTATCCATCGTAGTCTTGGTGGGCCGTTACCCCGCCAACAAACTAATGGAACGCATCCCCATCGATAACCGAAATTCTTTAATAAAAATATCATGCGATATTCGTATGCTATCCGG	428bp
